# Chimeric antigen receptor‐macrophages: A new paradigm for cell therapy

**DOI:** 10.1002/btm2.70157

**Published:** 2026-06-29

**Authors:** Heng Wang, Yaling Li, Yiwei Shi, Yun Zhou, Guoping Zheng

**Affiliations:** ^1^ Centre for Transplant and Renal Research, Westmead Institute for Medical Research, The University of Sydney Sydney New South Wales Australia; ^2^ Faculty of Medicine and Health The University of Sydney Camperdown New South Wales Australia; ^3^ Department of Vascular Surgery Beijing Anzhen Hospital, Capital Medical University Beijing China; ^4^ NHC Key Laboratory of Pneumoconiosis, Shanxi Key Laboratory of Respiratory Diseases Department of Pulmonary and Critical Care Medicine The First Hospital of Shanxi Medical University Taiyuan China; ^5^ Department of Nephrology The First Hospital of Shanxi Medical University Taiyuan Shanxi China

**Keywords:** CAR‐M, chimeric antigen receptor, fibrosis, inflammation, macrophages, solid tumor

## Abstract

Chimeric antigen receptor (CAR) technology has propelled CAR‐T cells to transformative success in hematologic malignancies, yet translation to solid tumors remains limited, motivating exploration of alternative CAR‐engineered immune effectors. Macrophages, sentinels of the innate immune system, are abundantly recruited to solid tumors, making them compelling candidates. Preclinical studies show that CAR‐engineered macrophages (CAR‐M) exhibit precise tumor homing, potent antigen‐directed phagocytosis, and the capacity to remodel immunosuppressive tumor microenvironments (TMEs). Notably, early‐phase clinical investigations indicate a favorable safety profile, strengthening confidence in their therapeutic potential against solid cancers. In this review, we synthesize design principles for CAR‐M constructs, with a particular focus on emerging engineering strategies for next‐generation CAR‐M. We further discuss their applications in oncology and emerging non‐oncologic indications, and summarize the current clinical trial landscape. We further propose a “4S framework” (specificity, switchability, synergy, and safety) to guide next‐generation CAR‐M development. Collectively, these advances support CAR‐M as a new paradigm for cancer therapy and beyond.

**Translational Impact Statement**

Chimeric antigen receptor‐engineered macrophages (CAR‐M) represent a next‐generation cellular therapy for solid tumors by overcoming key limitations of CAR‐T cells, including poor tumor infiltration and an immunosuppressive microenvironment. By leveraging macrophage phagocytosis, antigen presentation, and tumor microenvironment remodeling, CAR‐M therapy enables both direct tumor clearance and durable immune activation. These advances provide a translational framework for developing safer, more effective cell therapies for cancer and other macrophage‐driven diseases.

## 1 INTRODUCTION

Chimeric antigen receptors (CARs) are synthetic transmembrane receptors that, when introduced into immune cells, relay defined activation signals to execute specific effector functions. The flagship of this technology, CAR‐T cell therapy has produced transformative outcomes in hematologic malignancies, delivering substantial clinical benefit for B‐cell leukemias, lymphomas, and relapsed/refractory multiple myeloma.^1^, ^2^, ^3^ By contrast, translation to solid tumors has been disappointing.^4^ Relative to blood cancer, solid tumors display a more complex tumor microenvironment (TME): dense stromal architecture impedes lymphocyte trafficking and infiltration, while immunosuppressive mediators and cells blunt CAR‐T cytotoxicity.^5^ In addition, antigen heterogeneity and variable antigen specificity within solid tumors increase both the risk of immune escape and on‐target/off‐tumor toxicity.^6^ Although numerous engineering strategies are being pursued to optimize CAR‐T design, exploring alternative CAR‐engineered immune effectors represents a compelling and complementary path forward.^7^, ^8^

Macrophages are key innate immune effectors and are highly enriched within solid tumors. In the TME, diverse cues drive tumor‐associated macrophages (TAMs) toward immunosuppressive, pro‐tumor polarization states that facilitate growth and metastasis.^9^ Redirecting TAMs to pro‐inflammatory, anti‐tumor phenotypes is therefore an attractive therapeutic strategy.^10^ Beyond oncology, macrophages' intrinsic capacities for potent phagocytosis, tissue infiltration, and secretion of cytokines and chemokines position them as versatile agents in inflammation, repair, and regeneration.^11^ In 2006, Biglari et al. first transduced human primary monocytes with a carcinoembryonic antigen (CEA)–targeted CAR and demonstrated significant suppression of tumor growth in mice.^12^ Over the past 5 years, interest in CAR‐M has surged, with encouraging preclinical performance in both solid and hematologic tumor models^13^, ^14^ and expanding applications to inflammatory and fibrotic diseases^15^, ^16^ (Figure 1a,b). Notably, several early‐phase clinical studies (evaluating candidates such as MT‐101, SY001, and CT‐0508) have reported favorable safety profiles, underscoring the translational promise of CAR‐M.^17^, ^18^, ^19^, ^20^

**FIGURE 1:**
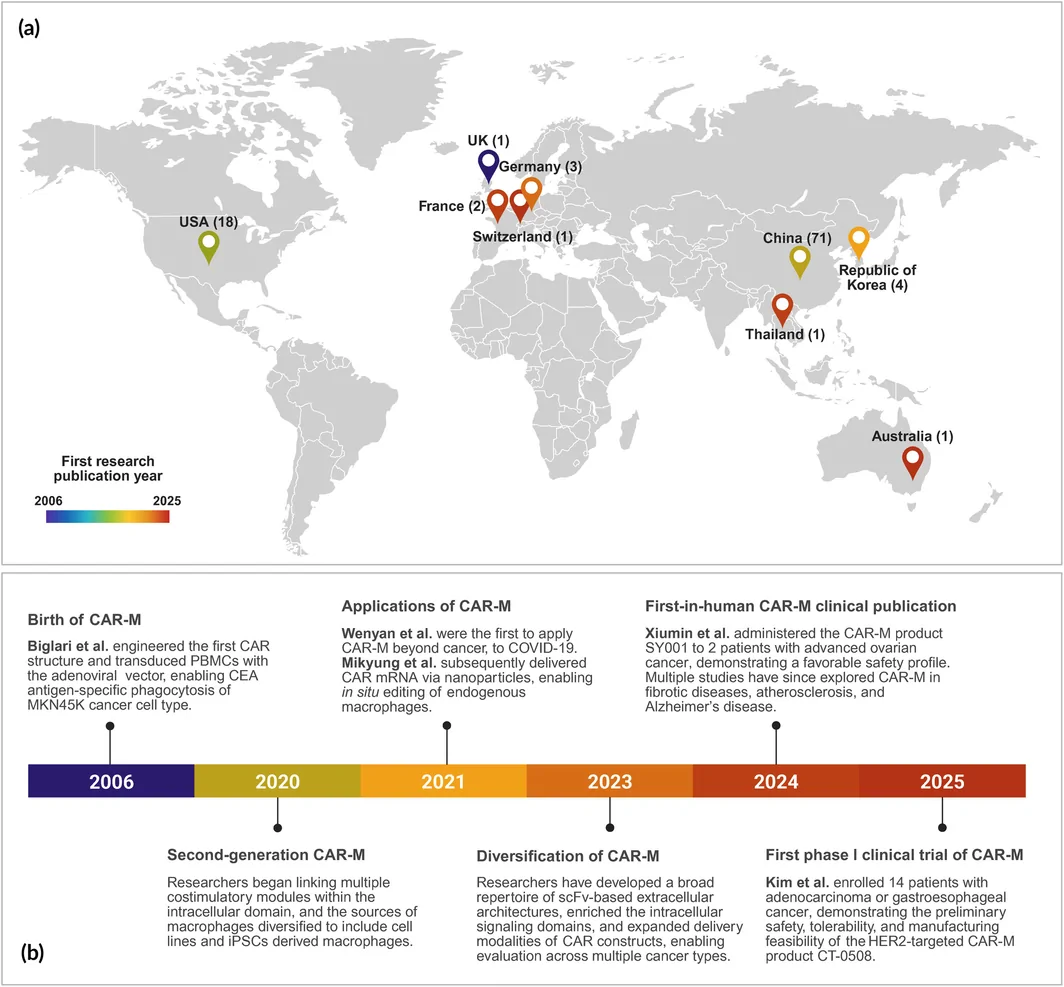
Distribution and key advances in CAR‐M therapy. (a) Global geographic distribution of CAR‐M therapeutic studies. (b) The timeline above illustrates each technology, each technology's journey from its initial design to current clinical trials, highlighting the year of major achievements such as initial successful CAR‐M design, technological iterations, applications of CAR‐M, and progression to clinical trials. Created with BioRender.com.

In this review we provide an in‐depth analysis of CAR‐M design principles, including CAR architecture, cell sources, and delivery strategies, with a particular focus on emerging engineering strategies for next‐generation CAR‐M as well as their preclinical advances and early clinical translation in oncology. We also outline emerging applications of CAR‐M beyond cancer and propose the “4S framework” (specificity, switchability, synergy, and safety) to guide the development of next generation CAR‐M therapies.

## 2 OVERVIEW OF CAR‐M DESIGN

### 2.1 CAR architecture

CARs are modular, artificially designed proteins that redirect engineered immune cells to specific targets and induce predefined signaling responses. A canonical CAR typically consists of an extracellular target‐binding domain, a hinge or spacer region, a transmembrane domain, and intracellular signaling modules. The extracellular antigen‐recognition domain is typically composed of a single‐chain variable fragment (scFv), which enables specific target recognition in a major histocompatibility complex (MHC)‐independent manner. The hinge region, commonly derived from IgG or CD8α, provides structural flexibility that facilitates antigen binding across different spatial conformations. The transmembrane domain is responsible for anchoring the CAR to the cell membrane and is often derived from CD3ζ, CD28, or CD8α. The intracellular signaling domain generally comprises activation and co‐stimulatory signaling modules that initiate downstream signaling cascades upon antigen engagement, thereby triggering macrophage phagocytic and cytotoxic functions.^21^ CAR‐M design largely draws on CAR‐T engineering. Over more than three decades, CAR‐T architectures have progressed through five generations of refinement.^21^, ^22^ Similarly, most current CAR‐M constructs correspond to first‐ or second‐generation formats (Figure 2).

**FIGURE 2:**
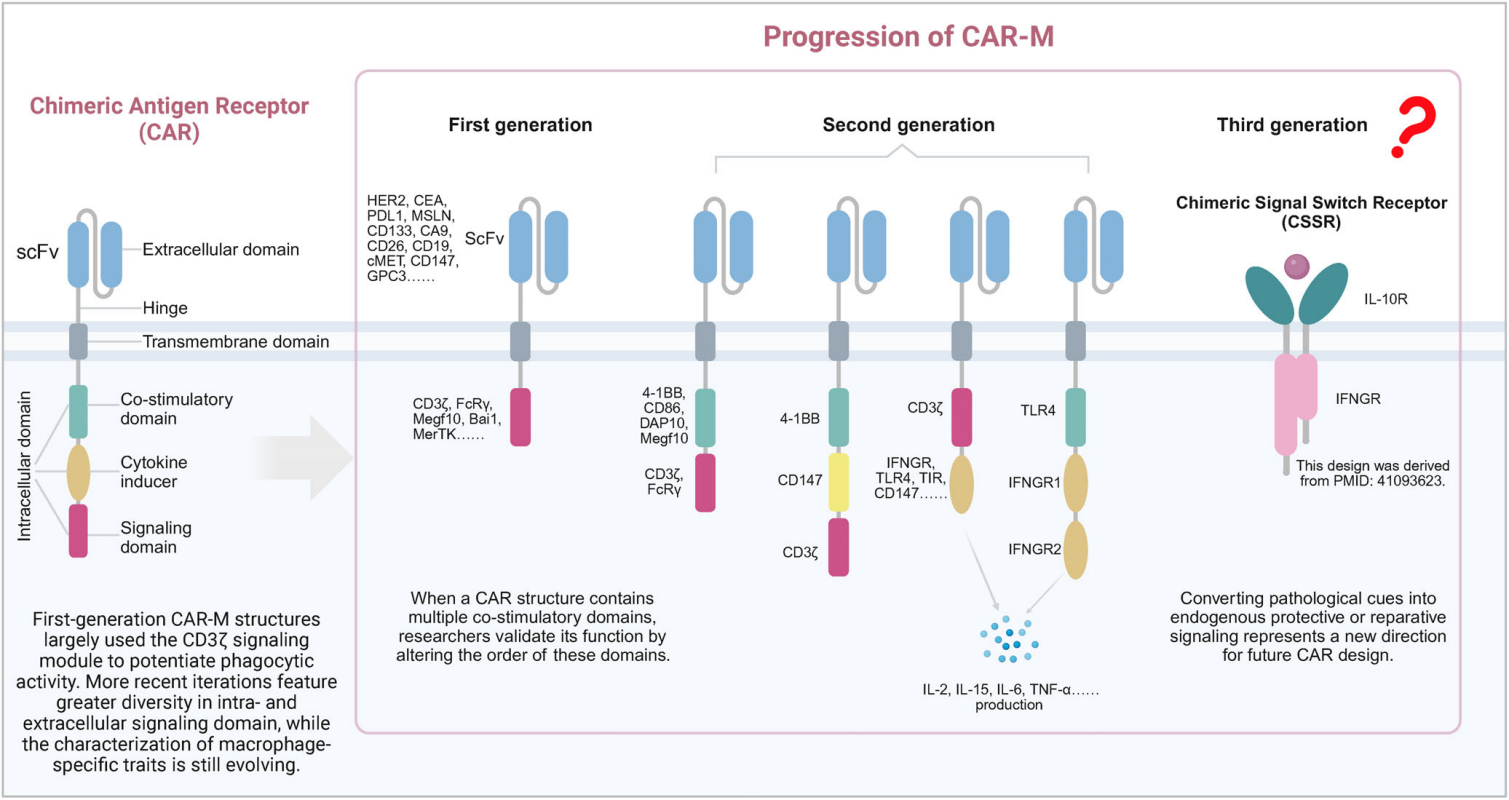
Components of CAR and iterative development of CAR‐M. CAR is composed of an extracellular antigen‐recognition domain, a hinge, a transmembrane domain, and an intracellular signaling domain (including co‐stimulatory domain, cytokine inducer, and signaling domain). Based on the evolution of the intracellular signaling domain, the iterative update of CAR‐M can be categorized into three generations: the first generation only contains the intracellular module harboring immunoreceptor tyrosine‐based activation motifs (ITAMs, e.g., CD3ζ, FcRγ etc.); the second generation integrates the co‐stimulatory domain or cytokine inducer on the basis of the first generation, and some only rely on dual cytokine‐inducing domains; and the third generation is designed based on chimeric cytokine receptor (ChCR), which enable precise molecular‐switch control over downstream signaling pathways. Created with BioRender.com.

In first‐generation CAR‐T constructs, the intracellular region contains only the CD3 zeta chain (CD3ζ) signaling domain, and the absence of costimulatory modules limits signal persistence and overall activity.^23^ By analogy, first‐generation CAR‐M employ a single intracellular module harboring immunoreceptor tyrosine‐based activation motifs (ITAMs), such as FcRγ, CD3ζ, or Megf10; antigen recognition leads to ITAM phosphorylation and yields relatively stable phagocytic activity.^24^, ^25^, ^26^ Second‐generation CAR‐M can be grouped into three strategies. The first integrates a costimulatory domain, for example, 4‐1BB, CD28, or DAP10, with CD3ζ to enhance phagocytosis.^27^, ^28^ The second combines CD3ζ with a cytokine‐inducing module, for example IFN‐γ, CD147, or TLR4, to couple target engagement with cytokine release.^29^, ^30^ The third replaces CD3ζ with dual cytokine‐inducing domains, such as TLR4 together with IFNGR1 and IFNGR2, to program stronger inflammatory activation.^31^

In addition, a recent study engineered a chimeric cytokine receptor (ChCR) that uses IL10RA/IL10RB or TGFβR1/TGFβR2 as the extracellular sensor and IFNGR1/IFNGR2 as the intracellular signaling module.^13^ This design embodies the concept of a chimeric signal‐switch receptor (CSSR) and represents a highly promising direction for third‐generation CAR‐M.

### 2.2 Cell sources for CAR‐M

In vitro engineering CAR‐M has relied on three principal sources: immortalized macrophage or monocyte cell lines, primary monocytes or macrophages, and pluripotent or progenitor cells. In addition, several studies have injected modified CAR directly into mice to achieve in situ editing of resident macrophages. Practical implementations by source are summarized in Tables 1, 2 and Figure 3.

**TABLE 1:** Preclinical studies of CAR‐M in cancer.

No.	Target disease	Extracellular domain	Intracellular domain	CAR delivery method	Cell source	Publication time	First author (country)	PMID
1	IL‐13Rα2‐positive solid tumors	Anti‐IL‐13Rα2 scFv	CD28 + CD3ζ	Lentiviral vector	iPSCs	2026	Ying Yang (China)	41983511^149^
2	Choroidal melanoma	Anti‐TYRP1 scFv	CD3ζ	Lipid nanoparticle	In situ autologous macrophages	2026	Wenfei Chen (China)	41960698^150^
3	Post‐surgery solid tumor recurrence	Anti‐IL‐13Rα2 scFv	NA	Lipofectamine 8000, 3D‐printed GelMA hydrogel implantation	RAW264.7, in situ autologous macrophages	2026	Dingmeng Nie (China)	41957823^151^
4	Prostate cancer	Anti‐PSMA scFv	CD3ζ	ZIF‐8‐based nanocarrier	BMDMs, in situ autologous macrophages	2026	Zichen Liang (China)	41950518^60^
5	T‐cell malignancy	Anti‐CD5 scFv	CD3ζ	Lentiviral vector	iPSCs	2026	Baoqiang Kang (China)	41943855^152^
6	Breast cancer	Anti‐HER2 scFv	CD28 + CD3ζ + SLC38A2	Adenoviral vector	PBMCs	2026	Mingzhu Liu (China)	41925028^153^
7	Ovarian and breast cancer	Anti‐HER2 scFv	Integrin α1β1 + FcγRI + GM‐CSF	Lentiviral vector	THP‐1, PBMCs	2026	Fuyu Du (China)	41856807^154^
8	Glioblastoma multiforme	SIGLEC9	TLR4 + CD40	Lipid nanoparticle	BMDMs, in situ autologous macrophages	2026	Zhipeng Fu (China)	41843671^155^
9	Prostate cancer	Anti‐hPSMA scFv	CD3ζ	Lipid nanoparticle	RAW264.7, BMDMs, in situ autologous macrophages	2026	Zhen Xu (China)	41819724^58^
10	Brain metastasis	Anti‐MSLN scFv	CD3ζ, MyD88	Lentiviral vector	BMDMs, PBMCs	2026	Shih‐Ying Wu (USA)	41772175^156^
11	Colorectal cancer	Anti‐GPA33 scFv	FcγRI + CD19	Lentiviral vector	THP‐1	2026	Daijun Wang (China)	41749316^157^
12	Prostate cancer	Anti‐NKG2D scFv	FcRγ + TLR4	Lentiviral vector	RAW264.7	2026	Rong Li (China)	41747445^158^
13	Solid tumor	Anti‐HER2 scFv, Anti‐CD19 scFv	4‐1BB + CD3ζ	Lentiviral vector	RAW264.7, BMDMs, in situ autologous macrophages	2026	Qimeng Yin (China)	41712635^159^
14	Heterogeneous glioblastoma	Anti‐sfGFP nanobody	IFNγR1 + CD3ζ	Virus‐mimetic targeting nanoplatforms	RAW264.7, BMDMs, in situ autologous macrophages	2026	Yulin Zhang (China)	41605215^160^
15	Pan‐cancer	Anti‐HER2 scFv	CD3ε, FCGR1A, FCGR2A, FCGR2B, or FCGR3A	Lentiviral vector	iPSCs	2026	Xinzhi Yang (China)	41602835^161^
16	CD19^+^ leukemia	Anti‐CD19 scFv	4‐1BB + CD3ζ	Electroporation	PBMCs	2026	Liangliang Liao (Germany)	41558599^162^
17	Solid tumor	Anti‐MSLN scFv	NA	Lentiviral vector	iPSCs	2026	Xuefei Wang (China)	41535541^163^
18	Peritoneal metastasis in solid tumors	scFv from Trastuzumab or mutated SIRPα	CD3ζ + TLR4	Lipid nanoparticle	RAW264.7, BMDMs, in situ autologous macrophages	2025	Kedan Gu (China)	41444487^68^
19	Ovarian cancer	Anti‐CD47 scFv	4‐1BB + CD3ζ + IL‐21	Adenoviral vector	THP‐1, PBMCs	2025	Yizhao Chen (China)	41436871^164^
20	Hepatocellular carcinoma	Anti‐NKG2D scFv	FcγRI	Lentiviral vector	BMDMs	2025	Zihao Zhao (China)	41388305^66^
21	Melanoma	Anti‐GP75 scFv	CD28 + CD3ζ	Lipid nanoparticle	BMDMs, in situ autologous macrophages	2025	JunHee Han (Republic of Korea)	41289602^55^
22	Pan cancer	Anti‐CD47 scFv	α1β1 integrin + FcγRI	Lentiviral vector	RAW264.7	2025	Fuyu Du (China)	41260905^165^
23	B receptor‐positive tumors	Anti‐RB4 scFv	CD28 + CD3ζ	Lentiviral vector	THP‐1	2025	Cyril Lherminier (France)	41222596^166^
24	Prostate cancer	Anti‐PSMA scFv	CD3ζ	Adenoviral vector	BMDMs, PBMCs	2025	Yangli (China)	41163070^64^
25	Triple‐negative breast cancer	IL10RA, IL10RB or TGFβR1, TGFβR2	IFNGR1, IFNGR2	pCWX Dest lentiviral vector	PBMCs, THP‐1	2025	Sabrina Traxel (Switzerland)	41093623^13^
26	Solid or diffuse tumors	Anti‐HER2 (4D5‐8) scFv	Fc receptor common gamma chain	Lentiviral vector	BMDMs	2025	Kirstin R. Rollins (USA)	41042177^33^
27	Mammary carcinoma	Anti‐HER2 scFv	NA	Lipid nanoparticle	BMDMs	2025	Jia Fu (China)	41009761^34^
28	Brest cancer	NA	NA	Ac_4_ManNAz treatment	RAW264.7, BMDMs	2025	Xin Ding (China)	40948141^92^
29	Colorectal cancer	Anti‐CEA scFv	CD3ζ or FcγRI	Lentiviral vector	Mouse myeloid progenitors	2025	Chuancheng Gao (China)	40847445^89^
30	Colon cancer and melanoma tumor	Anti‐PDL1 scFv	CD3ζ + IFN‐γ	Adenoviral vector	BMDMs	2025	Zhongbing Qi (China)	40814015^35^
31	Lung metastatic tumor	Anti‐MSLN scFv	NA	Small extracellular vesicles	BMDMs	2025	Yuchen Xiao (China)	40759657^50^
32	Pan cancer	Anti‐HER2 scFv	4‐1BB + CD3ζ	Lipid nanoparticle (circRNA)	In situ autologous macrophages	2025	Yanyan Wang (China)	40712575^106^
33	Glioblastoma multiforme	Anti‐CD133 scFv	CD3ζ + Toll/Il‐1R	Enucleated mesenchymal stem cells	RAW264.7, BMDMs, in situ autologous macrophages	2025	Lei Zhou (China)	40658861^44^
34	Ovarian cancer	Anti‐EphA2 scFv, anti‐CD19 scFv	FcGRIIA	Ad5f35 adenoviral vector	THP‐1, PBMCs	2025	Tianyi Liu (USA)	40595560^167^
35	Pancreatic ductal adenocarcinoma	Anti‐FAP scFv	CD3ζ	Lipid nanoparticle	BMDMs, in situ autologous macrophages	2025	Wenguang Wang (China)	40425095^97^
36	Pan cancer	Anti‐HER2 scFv	FcER1G + CD3ζ	Lentiviral vector, adenoviral vector	THP‐1, BMDMs	2025	Siqi Chen (USA)	40307254^49^
37	Renal cell carcinoma	Anti‐CA9 scFv, IL‐2R	CD3ζ, TLR4	Lipid nanoparticle (circRNA)	In situ autologous macrophages	2025	Weiqiang Jing (China)	40301655^69^
38	Melanoma	Anti‐CSPG4 scFv	FcRγ	Lentiviral vector	PBMCs	2025	Daniel Greiner (USA)	40082557^168^
39	Chronic myeloid leukemia	Anti‐CD26 scFv	4‐1BB + CD3ζ	Lentiviral vector	THP‐1, RAW264.7	2025	Jiang Guoyun (China)	39948620^14^
40	Breast cancer and gastroesophageal cancer	Anti‐HER2 scFv	CD3ζ	Ad5f35 adenoviral vector	PBMCs	2025	Kim A Reiss (USA)	39920391^20^
41	HER2+ solid tumors	Anti‐HER2 scFv	CD3ζ	Ad5f35 adenoviral vector	BMDMs	2025	Stefano Pierini (USA)	39814734^94^
42	Cancer	Anti‐CD19 scFv, anti‐cMet scFv	CD3ζ, 28ζ, Megf10, FcεRIA, FcγRIA, MerTK	Lentiviral vector	PBMCs	2025	Ji U Choi (Republic of Korea)	39748414^90^
43	Pancreatic ductal adenocarcinoma	Anti‐cMET scFv	FcRγ signaling activation domain, and the CD19 phosphoinositide 3‐kinase (PI3K) recruitment domain	Lentiviral vector, Ad5f35 adenoviral vector	THP‐1, BMDMs, PBMCs	2024	Huaijin Zheng (China)	39643883^169^
44	Breast cancer	Anti‐HER2 scFv	CD3ζ	Lentiviral vector, Ad5f35 adenoviral vector	THP‐1, PBMCs	2024	Lydia Ziane‐Chaouche (France)	39632807^63^
45	Cancer	Anti‐CD19 scFv	NA	Lipid nanoparticle	In situ autologous macrophages	2024	Alvin J Mukalel (USA)	39628840^132^
46	HER2+ solid tumors	Anti‐HER2 scFv	α1β1 integrin + FcγRI	pCDH lentiviral vector	J774A.1, THP‐1	2024	Fuyu Du (China)	39617174^32^
47	Ovarian cancer	Anti‐MSLN scFv	4‐1BB + CD3ζ	Ad5f35 adenoviral vector	PBMCs	2024	Xiumin Li (China)	39609867^18^
48	Pancreatic cancer	Anti‐cMET scFv	FcγRI + CD19	Lentiviral vector	Induced pluripotent stem cells (iPSCs)	2024	Lingyu Hu (China)	39592929^99^
49	Ovarian cancer	Anti‐HER2 scFv	CD3ζ	Ad5f35 adenoviral vector	PBMCs, RAW264.7	2024	Qinyao Zhu (China)	39390143^136^
50	Solid tumors	Anti‐HER2 scFv	DAP10 + CD3ζ, Megf10 + CD3ζ	hH11 sgRNA‐Cas9 plasmid	iPSCs	2024	Xing Zhen (Republic of Korea)	39383607^28^
51	Pan cancer	Anti‐P1h3 scFv, anti‐FMC63 scFv	FcRy, FcγRIIa, FcγRIIc	Lentiviral vector, Ad5f35 adenoviral vector	THP‐1	2024	Han Zhang (China)	39379603^145^
52	Cancer	Anti‐CD19 scFv	4‐1BB + CD3ζ	Lentiviral vector	THP‐1	2024	Yunhan Jiang (China)	39041307^95^
53	Breast cancer	Anti‐CD147 (M6‐1B9) scFv	FcRy	Lentiviral vector	THP‐1	2024	Koollawat Chupradit (Thailand)	38954079^170^
54	Hepatocellular carcinoma	Anti‐GPC3 scFv	CD3ζ	Ad5f35 adenoviral vector	RAW264.7	2024	Lili Guan (China)	38881757^171^
55	Brest cancer	Anti‐PD‐1	CD40, CD3ζ	Lentiviral vector	THP‐1, PBMCs	2024	Kayla Myers Chen (USA)	38726005^172^
56	Pan cancer	Anti‐CD19 scFv	CD3ζ, FcRγ, FcRγ + CD3ζ, IFNGR1 + CD3ζ, TLR4‐CD3ζ, TRAF4‐CD3ζ	Lentiviral vector	iPSCs, THP‐1	2024	Jun Shen (China)	38723634^29^
57	Pancreatic cancer	Anti‐PSCA scFv	CD3ζ + mIL15 + tEGFR	piggyBac vector	CD34+ hematopoietic stem and progenitor cells, iPSCs, THP‐1	2024	Zahir Shah (USA)	38663406^37^
58	HER2+ tumors	Anti‐HER2 Fab	CD3ζ + CD147, CD147 + CD3ζ, 4‐1BB + CD3ζ + CD147, 4‐1BB + CD147 + CD3ζ, CD147 + 4‐1BB + CD3ζ	Lentiviral vector	THP‐1	2024	Bing Yang (China)	38243698^30^
59	Leukemia	Anti‐CD19 scFv	FcεRI + CD19	Lentiviral vector	Primary hematopoietic stem and progenitor cells, iPSCs	2023	Shifaa M Abdin (Germany)	38135346^38^
60	Cancer	Anti‐CD19 scFv	NA	Lentiviral vector	BMDMs	2023	Abhinava K Mishra (USA)	38109551^173^
61	Cancer	Anti‐HER2 scFv	CD3ζ, CD32a	Lentiviral vector	RAW264.7, PBMCs	2023	Yun Gao (China)	38058430^48^
62	Pan cancer	Anti‐EGFRvIII scFv, anti‐GPC3 scFv	CD3ζ, TIR, CD3ζ + TIR	Lentiviral vector	iPSCs	2023	Anhua Lei (China)	38012418^39^
63	Ovarian cancer	Anti‐HER2 scFv, anti‐CD47 scFv	4‐1BB + CD3ζ	Adenoviral vector	THP‐1	2023	Yizhao Chen (China)	37740183^174^
64	Pan cancer	Anti‐MSLN scFv	CD3ζ	Lentiviral vector	iPSCs	2023	Xudong Wang (China)	37723178^62^
65	Solid tumors	Anti‐MSLN scFv	NA	Lentiviral vector	iPSCs	2023	Jicheng Wu (China)	37695897^175^
66	Hepatocellular carcinoma	Anti‐GPC3 scFv	CD3ζ	Lipid nanoparticle	RAW264.7, BMDMs, THP‐1, in situ autologous macrophages	2023	Zhenmei Yang (China)	37451547^56^
67	Breast cancer	Anti‐VEGFR2 scFv	Tlr4 + Ifngr1 + Ifngr2	Lentiviral vector	RAW264.7	2023	Zhaojun Duan (China)	37438548^31^
68	Gastric cancer	Anti‐HER2 scFv	FcεR1γ	Lentiviral vector	Primary peritoneal macrophages	2023	Xuhui Dong (China)	37386139^101^
69	Pan cancer	Anti‐HER2 scFv	FcεR1γ	Lentiviral vector	BMDMs	2023	Yi Huo (China)	36978075^93^
70	Brainstem glioma	Anti‐ErbB2 scFv	CD3ζ	DNA nanocarriers	BMDMs, RAW264.7	2023	Lin Gao (China)	36805678^91^
71	Pan cancer	Anti‐GD2 scFv	CD3ζ	CRISPR‐Cas9	iPSCs	2023	Jue Zhang (USA)	36638788^51^
72	Pan cancer	Anti‐CD19 scFv	FcRγ, PI3K, Megf10	Lentiviral vector	BMDMs	2022	Maoxuan Liu (China)	36429120^105^
73	Glioblastoma multiforme	Anti‐CD133 scFv	CD3ζ	Nanoparticles‐hydrogel superstructure	THP‐1, BMDMs, in situ autologous macrophages	2022	Chen Chen (China)	35921473^104^
74	Pan cancer	Anti‐CEA scFv	CD3ζ	Lentiviral vector	Primary human hematopoietic stem and progenitor cells	2022	Daniela Paasch (Germany)	35326445^36^
75	B lymphoma	Anti‐CD19 scFv	NA	Lipid nanoparticle	RAW264.7, BMDMs, in situ autologous macrophages	2022	Zhongfeng Ye (USA)	35104103^176^
76	Solid tumors	Anti‐ALK scFv	CD3ζ + IFN‐γ	Nanoparticles	RAW264.7, BMDMs, in situ autologous macrophages	2021	Mikyung Kang (Republic of Korea)	34510559^59^
77	Pan cancer	Anti‐CD19 scFv	4‐1BB + CD3ζ, CD86 + FcγRI	Lentiviral vector	iPSCs	2020	Li Zhang (China)	33176869^27^
78	Breast cancer	Anti‐CCL19 Fab	TLR2, TLR4, TLR6, MerTK, 4‐1BB + CD3ζ	Lentiviral vector	RAW264.7	2020	Zhiyuan Niu (China)	33140856^96^
79	Pan cancer	Anti‐CD19 scFv, Anti‐HER2 scFv	CD3ζ, FcRy	Lentiviral vector, Ad5f35 adenoviral vector	THP‐1	2020	Michael Klichinsky (USA)	32361713^26^
80	Breast cancer	Anti‐HER2 scFv	CD147	Lentiviral vector	RAW264.7	2019	Wenlong Zhang (China)	31570753^98^
81	Pan cancer	Anti‐CD19 scFv	Megf10, FcRγ, Bai1, MerTK	Lentiviral vector	BMDMs	2018	Meghan A Morrissey (USA)	29862966^25^
82	CEA‐positive tumor	Anti‐CEA scFv	FcγRI	Adenoviral vector	PBMCs	2006	A Biglari (UK)	16397508^12^

**TABLE 2:** Preclinical studies of CAR‐M in non‐cancer diseases.

No.	Target disease	Extracellular domain	Intracellular domain	CAR delivery method	Cell source	Publication time	First author (country)	PMID
1	Renal fibrosis	Anti‐FAP scFv	CD137 + CD3ζ + IL‐4	Lentiviral vector	THP‐1	2026	Wenyan Zhao (China)	41887221^177^
2	Systemic amyloidosis	Pan‐amyloid reactive peptide p5	CD3ζ	Lentiviral vector	THP‐1	2026	Manasi Balachandran (USA)	41869320^178^
3	Liver fibrosis	Anti‐FAP scFv	CD3ζ	Lipid nanoparticle	RAW264.7, BMDMs, in situ autologous macrophages	2026	Jinxin Gao (China)	41760658^179^
4	Alzheimer's disease	Anti‐AβO scFv	CD3ζ	Lipid nanoparticle	In situ autologous microglia	2026	Chongzheng Yan (China)	41702507^180^
5	Pulmonary fibrosis	Anti‐FAP scFv	4‐1BB + CD3ζ	Lentiviral vector	THP‐1, RAW264.7	2026	Yan Zhang (China)	41688427^181^
6	Liver and lung fibrosis	Anti‐FAP scFv	CD3ζ	Adenoviral vector	RAW264.7, BMDMs	2026	Min Wang (China)	41578643^182^
7	Liver fibrosis	Anti‐TNC scFv	4‐1BB + CD3ζ	Lentiviral vector	RAW264.7, BMDMs	2025	Kaizhao Chen (China)	41214839^183^
8	Myocardial ischemia–reperfusion injury	Anti‐FAP scFv	CD147	Lipid nanoparticles	Cardiac macrophages isolated from mouse	2025	Heng Du (China)	40762067^16^
9	Acute and chronic inflammatory diseases	Anti‐TNF scFv	IL‐4Rα	Lentiviral vector	BMDMs, PBMCs	2025	Qi Cao (Australia)	40335685^15^
10	Myocardial infarction	Anti‐FAP scFv	CD3ζ	Lipid nanoparticles	RAW264.7, BMDMs, in situ autologous macrophages	2025	Zejuan Liu (China)	40223483^42^
11	Myocardial ischemia–reperfusion injury	Anti‐FAP scFv	CD147	Lentiviral vector	BMDMs	2024	Jiawan Wang (China)	39465245^119^
12	Skin scar	Anti‐DPP4 scFv	CD3ζ	Lentiviral vector	BMDMs	2024	Min Liu (China)	39313983^120^
13	Alzheimer's disease	Anti‐Aβ scFv	FcRγ	Retroviral vector	BMDMs	2024	Alexander B Kim (USA)	38516884^65^
14	Atherosclerosis	Anti‐CD47 scFv	CD3ζ	Lentiviral vector	THP‐1	2024	Skylar T Chuang (USA)	38353580^126^
15	Liver fibrosis	Anti‐uPAR scFv	CD3ζ	Adenoviral vector	BMDMs	2024	Hanren Dai (China)	38340812^121^
16	Sepsis	Anti‐SasA	NA	Lipid nanoparticles	RAW264.7, BMDMs, in situ autologous macrophages	2024	Chunwei Tang (China)	38207332^57^
17	Myocardial ischemia–reperfusion injury	NA	CCR2, MerTK overexpression	Adenoviral vector	BMDMs	2024	Haipeng Tan (China)	38198534^184^
18	Periprosthetic joint infection	Anti‐SasA scFv	CD3ζ	Peptide nanoparticles	RAW264.7, BMDMs	2023	Ziyang Li (China)	37256944^109^
19	Inflammation‐related depression	Anti‐CTLA‐4	NA	Liposomes	RAW264.7	2022	Yu Liu (China)	34897839^43^
20	Coronavirus disease 2019 (COVID‐19)	Anti‐CR3022 scFv	Fcγ, MEGF10, MERTK, CD3ζ	Lentiviral vector	THP‐1	2021	Wenyan Fu (China)	34367135^110^

**FIGURE 3:**
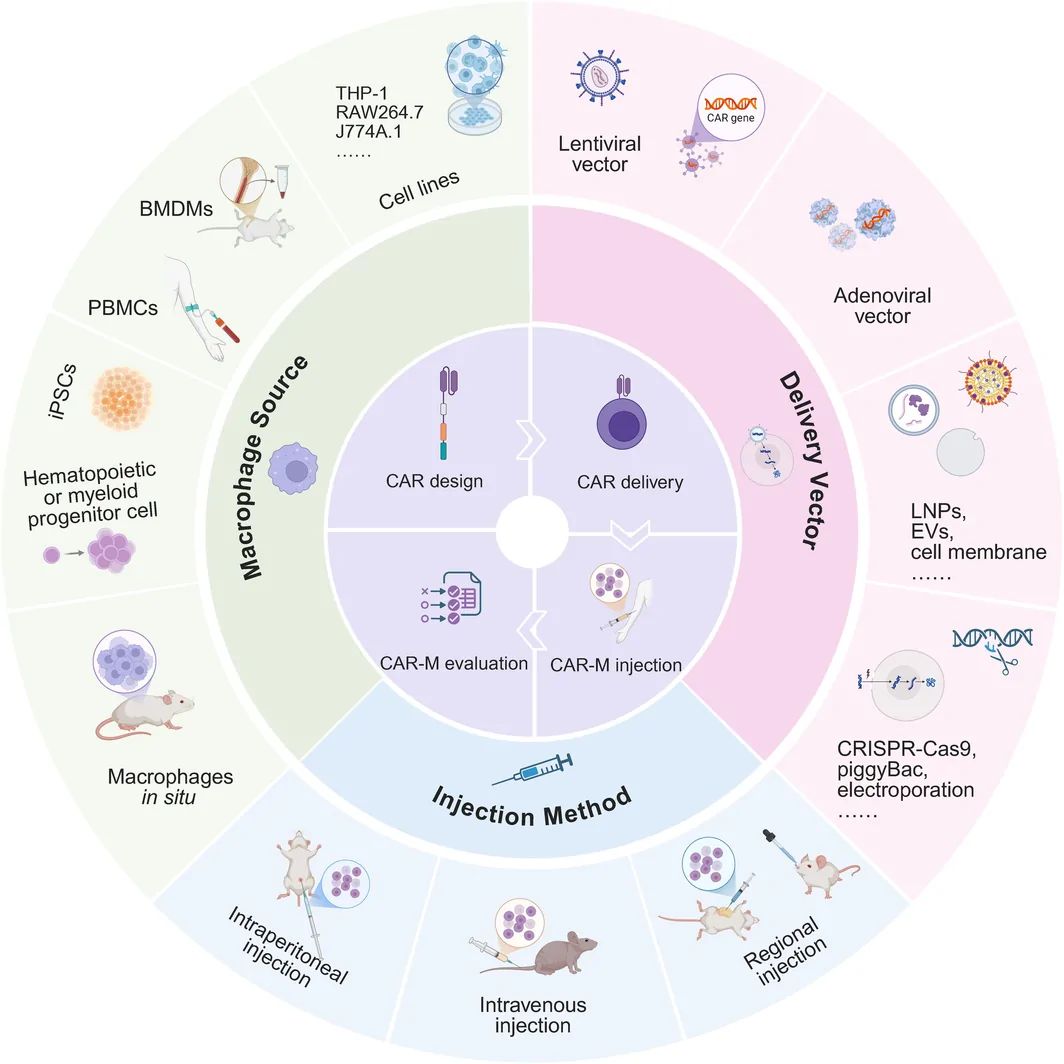
Key points of CAR‐M therapy. Overview of core elements of CAR‐M therapy, including macrophage sourcing, gene delivery vectors, and administration methods. Created with BioRender.com.

Macrophage cell lines are commonly used sources of CAR‐M for in vitro studies and validation in mouse models, including RAW264.7, THP‐1, and J774A.1 cells.^14^, ^32^ Cell lines offer several advantages as CAR‐M sources. They are readily accessible. After CAR transfer, CAR‐positive cells can be purified by flow cytometric sorting. Purified CAR‐M can be expanded at scale while maintaining a stable CAR phenotype.

Primary cell‐based CAR‐M can improve autologous compatibility and safety. In diverse mouse models, bone marrow‐derived macrophages (BMDM) are a key source for CAR‐M.^33^, ^34^, ^35^ After harvest of mouse bone marrow and differentiation with M‐CSF, the resulting macrophages are typically terminally differentiated, which helps mitigate the risk of uncontrolled proliferation in vivo. Limited source material and relatively low viral transduction efficiency of the CAR construct, approximately 40%, remain major constraints on BMDM applications.^15^ In clinical trials of CAR‐M, autologous peripheral blood mononuclear cells (PBMC) constitute the main source of macrophages.^18^, ^20^ Participants first received granulocyte colony‐stimulating factor to mobilize peripheral hematopoietic cells (4–5 days); PBMCs were then collected, and CD14^+^ monocytes were isolated; granulocyte–macrophage colony‐stimulating factor (GM‐CSF) was added to differentiate them into macrophages (3–5 days), and the Ad5/f35 adenoviral vector was used to transfer the CAR transgene into macrophages (2–3 days). This workflow completes CAR‐M manufacturing in about 9 to 13 days. For the CT‐0508 product, the mean cell viability was 86.39%, the purity was 86.78%, and the CAR transduction efficiency was 79.28%.^20^

Primary hematopoietic stem and progenitor cells (HSPC) and induced pluripotent stem cells (iPSC) offer strong solutions to the scarcity of mature macrophage sources. CAR expression has been achieved in cord blood‐derived CD34^+^ HSPCs, which display substantial expansion potential during differentiation into CAR‐M.^36^, ^37^, ^38^ iPSCs provide even broader sourcing options. They can be reprogrammed from CD34^+^ HSPCs,^37^ obtained as commercial cell products,^28^ or generated from bone marrow and peripheral blood cells.^29^, ^39^ Scalable macrophage production from iPSCs is expected to accelerate clinical translation of CAR‐M therapies.^29^, ^40^, ^41^

Finally, biomaterials supported CAR delivery systems have been shown to edit macrophages in situ at disease sites in mouse models, enabling local generation of CAR‐M within target tissues.^16^, ^42^, ^43^, ^44^

### 2.3 Delivery of CAR constructs

Viral vectors (lentivirus and adenovirus) are the most used systems for introducing CAR transgenes into macrophages. Because macrophages exhibit innate resistance to virus‐mediated gene transfer, high vector titers are often required, which raises concerns about cytotoxicity and suboptimal transduction efficiency.^45^, ^46^, ^47^ Yun et al. improved lentiviral delivery by packaging the HIV‐2 accessory protein Vpx into an HIV‐1‐derived system, achieving approximately 70% transduction in macrophages differentiated from PBMCs.^48^ Adenoviral Ad5/F35 is another efficient platform. Two clinical studies reported transduction efficiencies of up to 80% in macrophages derived from human PBMCs.^18^, ^20^ Viral transduction can also activate inflammasomes and thereby enhance the proinflammatory phenotype and phagocytic capacity of CAR‐M.^35^, ^49^

Among nonviral vectors, engineered nanoparticles and biomembrane‐based carriers are commonly used and leverage biocompatibility to deliver CAR into macrophages.^34^, ^43^, ^50^ These platforms can support in situ editing of macrophages and largely circumvent the cargo size limitations of viral vectors, which enables exploration of synergistic payloads. Finally, other approaches such as CRISPR–Cas9, piggyBac vectors, and electroporation have also been reported for CAR delivery.^28^, ^37^, ^51^, ^52^

## 3 EMERGING ENGINEERING STRATEGIES FOR NEXT‐GENERATION CAR‐M

### 3.1 In vivo and in situ generation strategies for CAR‐M

#### 3.1.1 Lipid nanoparticles delivery

Lipid nanoparticles (LNPs), as a well‐established delivery platform, have been extensively investigated and broadly applied over the past several decades. Typically composed of phospholipids, cholesterol, and surfactants, LNPs enable the efficient encapsulation and targeted delivery of nucleic acid therapeutics and are currently recognized as one of the most clinically non‐viral RNA delivery systems.^53^, ^54^ Their advantages, including relatively simple manufacturing procedures, favorable biocompatibility, and compatibility with repeated administration, make them highly promising vehicles for gene therapy.

In recent years, LNPs have been increasingly utilized for the in vivo targeted transfection of TAMs, thereby circumventing complex ex vivo manipulation procedures and directly inducing CAR expression within the TME. This strategy has been shown to markedly enhance macrophage phagocytic and cytotoxic activities while simultaneously promoting local antitumor immune responses, thereby effectively suppressing tumor progression. For example, PS‐LNPs, ARA‐LNPs, and CAR&Siglec‐GΔITIMs LNPs have all demonstrated potent antitumor efficacy.^34^, ^55^, ^56^ Beyond cancer therapy, LNPs‐mediated in vivo CAR‐M engineering has also been extended to other disease settings. For instance, in myocardial ischemia–reperfusion injury and methicillin‐resistant *Staphylococcus aureus* (MRSA) infection, LNP‐FAP CAR and CRV/LNP‐RNAs have been employed to generate functionally enhanced CAR‐M in vivo, thereby alleviating myocardial fibrosis, overcoming intracellular immune evasion by pathogens, and improving disease outcomes.^16^, ^57^

Moreover, the delivery performance of LNPs can be further enhanced through structural optimization of lipid components, such as the incorporation of fluorinated ionizable lipids.^58^ Nevertheless, several challenges remain for LNPs‐based delivery systems, including insufficient in vivo stability, potential cytotoxicity, and immunogenicity. Therefore, future studies should focus on improving stability, minimizing toxicity, and optimizing delivery efficiency to facilitate the clinical translation of LNPs‐mediated CAR‐M therapies.

#### 3.1.2 Other delivery systems

In addition to the strategies described above, several emerging non‐viral delivery systems have demonstrated considerable promise for CAR‐M engineering. For example, a non‐viral piggyBac transposon system has been developed in which macrophage‐targeting nanocarriers and plasmid DNA encoding CAR‐IFN‐γ are delivered in vivo to achieve in situ macrophage reprogramming. This approach promotes the polarization of macrophages toward a CAR‐M1 phenotype with enhanced phagocytic and immunomodulatory functions, thereby suppressing solid tumor growth.^59^ Engineered small extracellular vesicles (sEVs) incorporating anti‐CD206 scFvs have also been shown to selectively target M2 macrophages and induce their conversion toward an antitumor phenotype, resulting in effective tumor suppression and enhanced long‐term immune memory.^50^ In addition, metal–organic framework (MOF)‐based nanosystems constructed from zeolitic imidazolate framework‐8 (ZIF‐8) exhibit excellent gene‐loading capacity and efficient targeted delivery to TAMs, thereby enhancing macrophage‐mediated phagocytic and cytotoxic activities against tumor cells while simultaneously promoting adaptive immune activation.^60^

Recently, biomimetic delivery strategies have also achieved substantial progress. For example, the erythrocyte‐based mRNA‐LNP‐Ery platform has enabled the in vivo generation of CAR‐engineered myeloid cells for cancer immunotherapy.^61^ Furthermore, enucleated mesenchymal stem cells (eMSCs), which retain intrinsic tumor‐homing properties, have been employed as delivery carriers to enable in situ CAR‐M transfection and remodel the tumor immune microenvironment.^44^

### 3.2 Synthetic and modular intracellular signaling domains

CAR‐M technology is derived from the CAR‐T platform and generally consists of four major components: an extracellular antigen‐recognition domain, a hinge region, a transmembrane domain, and an intracellular signaling domain. This modular architecture provides CARs with substantial engineering flexibility and programmability, enabling the rational combination of CAR constructs with distinct functional properties and preconditioned macrophages to generate customized CAR‐M systems tailored to specific disease contexts and therapeutic objectives.

Based on differences in intracellular signaling architectures, CAR‐M can be broadly categorized into three generations (Table 3). First‐generation CAR‐M constructs contain only a single activation signaling domain, such as CD3ζ, FcRγ, or Megf10. Although these constructs are capable of inducing phagocytosis, their antitumor efficacy and persistence remain relatively limited.

**TABLE 3:** Generations and functional features of CAR‐M systems.

Generation	Core structure	Intracellular signaling domains	Primary functions	Limitations
First	ScFv + hinge + TM + single intracellular signaling domain	CD3ζ, FcRγ, Megf10	Phagocytic and cytotoxic activity	Limited therapeutic efficacy and short persistence
Second	ScFv + hinge + TM + dual intracellular signaling domains	CD3ζ + 4‐1BB/CD28/DAP10	Enhanced phagocytic and cytotoxic activity	Limited controllability and potential off‐target effects
		CD3ζ + IFN‐γ/TLR4/TIR/CD147/Toll/IL‐1R	Antitumor activity and proinflammatory responses	
	ScFv + hinge + TM + multiple intracellular signaling domains	CD3ζ + 4‐1BB + CD147	Enhanced antitumor and proinflammatory activities	
		TLR4 + IFNGR1/IFNGR2	Sustained inflammatory activation and antitumor activity	
Third	Extracellular domains (IL‐10/TGF‐β) + intracellular signaling domains	IFN‐γ receptor‐associated signaling domains	Antitumor and proinflammatory activities with signal reprogramming capability	‐

To enhance functional potency, second‐generation CAR‐M incorporates additional co‐stimulatory signaling domains, including 4‐1BB, CD28, and DAP10, which markedly improve macrophage phagocytic and tumoricidal activities. Moreover, integration of inflammation‐associated signaling modules can further amplify proinflammatory cytokine secretion and enhance antitumor responses. For example, Duan et al. incorporated TLR4 and/or IFN‐γ receptor components (IFNGR1 and IFNGR2) into the intracellular signaling domain, successfully inducing TNF‐α secretion and sustained antitumor activity.^31^

Third‐generation CAR‐M further introduces signal “reprogramming” strategies through the construction of ChCRs to achieve context‐dependent functional modulation. For instance, fusion of the extracellular domains of IL‐10 or TGF‐β receptors with the intracellular domain of the IFN‐γ receptor enables macrophages to acquire a proinflammatory phenotype within the TME, thereby enhancing local antitumor immune responses while reducing systemic off‐target effects.^13^ In another study, a CAR incorporating an anti‐TNF scFv extracellular domain linked to the intracellular domain of IL‐4Rα was engineered to dynamically regulate inflammatory responses through signal conversion. Such designs render CAR‐M activity dependent on the local inflammatory milieu, thereby improving targeting precision while minimizing the risk of organ toxicity associated with sustained activation.^15^

Collectively, engineering strategies based on signal modulation and microenvironment‐responsive regulation provide CAR‐M systems with built‐in regulatory capabilities and may represent an important direction for the development of next‐generation cell‐based immunotherapies.

### 3.3 Signal programming and functional reprogramming of CAR‐M

First‐generation CAR‐M was structurally adapted from CAR‐T platforms and primarily incorporated the CD3ζ activation domain to mediate phagocytic activity. However, this signaling configuration is insufficient to sustain stable macrophage polarization or prolonged immune activation, thereby limiting overall antitumor efficacy.^20^, ^27^ To overcome these limitations, second‐generation CAR‐M has undergone substantial structural and functional optimization, enabling enhanced phagocytosis, sustained proinflammatory activation, and tissue remodeling capabilities, thereby improving their adaptability to the highly immunosuppressive and heterogeneous TME.

#### 3.3.1 Macrophage polarization programming

To enhance the durability of CAR‐M‐mediated antitumor activity, Lei et al. engineered a second‐generation CAR‐M system by fusing the CD3ζ domain with the toll‐like receptor 4 intracellular toll/interleukin‐1 receptor (TIR) signaling domain. This design not only significantly enhanced phagocytic activity but also suppressed M2 polarization through NF‐κB‐dependent signaling, thereby maintaining a sustained proinflammatory state and improving antitumor efficacy.^39^ In addition, macrophage functional states are tightly regulated by intracellular metabolic networks. Targeting key metabolic regulators and signaling pathways, including glycolytic activity, proprotein convertase furin, and the ACOD1/KEAP1/NRF2 axis, has been shown to induce metabolic reprogramming of macrophages, thereby promoting persistent inflammatory activation and enhancing tumoricidal and phagocytic functions.^62^, ^63^, ^64^ The integration of gene‐editing technologies with CAR engineering may therefore provide a promising strategy for precise macrophage phenotype remodeling and further optimization of CAR‐M therapeutic efficacy.

#### 3.3.2 Cytokine‐secreting CAR‐M

To further exploit the immunomodulatory potential of CAR‐M, cytokines or secretory proteins have been incorporated into CAR designs, enabling engineered macrophages to actively secrete cytokines and chemokines that modulate the local immune microenvironment.^22^ For example, Kim et al. developed a next‐generation CAR‐M capable of secreting macrophage colony‐stimulating factor (M‐CSF). In addition to target recognition, this engineered CAR‐M significantly reduced local amyloid plaque deposition through cytokine secretion.^65^ Moreover, activation of signaling pathways such as PI3K‐AKT and cGAS‐STING has been shown to enhance the secretion of chemokines including CXCL9, CXCL10, and CCL5, which are critical for lymphocyte recruitment and immune activation.^13^, ^66^

#### 3.3.3 Tissue remodeling functions

The immunosuppressive nature of the TME remains a major barrier limiting the therapeutic efficacy of CAR‐T and CAR‐NK therapies.^67^ In contrast, CAR‐M possesses intrinsic tissue‐infiltrating and phagocytic properties that confer unique advantages in remodeling the TME. Engineered CAR‐M can secrete matrix metalloproteinases (MMPs) to degrade extracellular matrix (ECM) components, thereby reducing physical barriers and improving immune cell infiltration. In addition, CAR‐M can interact with T cells, NK cells, and other immune populations within the TME to promote adaptive immune activation and enhance NK‐cell cytotoxicity, collectively contributing to the reprogramming of the immunosuppressive tumor milieu and providing new opportunities for solid tumor immunotherapy.^37^, ^68^, ^69^

#### 3.3.4 Inflammation modulation

Macrophages are key regulators of inflammation and tissue homeostasis and can polarize into either proinflammatory M1 or anti‐inflammatory M2 phenotypes in response to distinct microenvironmental cues.^70^, ^71^ In solid tumors, CAR‐M systems are typically engineered toward an M1‐like proinflammatory phenotype to exert antitumor activity through enhanced phagocytosis, antigen presentation, and inflammatory cytokine release.^72^ In contrast, in inflammatory disease settings, CAR‐M can be reprogrammed toward anti‐inflammatory phenotypes through recognition of inflammatory mediators such as TNF and activation of IL‐4 signaling pathways, thereby demonstrating therapeutic potential in both acute and chronic inflammatory disorders.^15^ Notably, these functional differences in macrophage polarization and effector responses are largely determined by variations in the intracellular signaling domains incorporated into CAR constructs.

#### 3.3.5 Tunable signaling strength

Engineering the CAR architecture also enables precise modulation of CAR‐M signaling intensity and functional activity. However, excessive activation and sustained signaling output may also introduce safety concerns, including cytokine release syndrome (CRS) and off‐target toxicity.^8^, ^73^ Therefore, further optimization of CAR‐M engineering strategies and safety‐control systems will be essential to facilitate their clinical translation.

### 3.4 Programmable and context‐dependent CAR‐M systems

Regulation of macrophage polarization states is critical for balancing therapeutic efficacy and safety in CAR‐M‐based immunotherapy. The high plasticity of macrophages represents both a major advantage and a potential challenge. Accordingly, CAR design must account for the ability of CAR‐M to maintain an antitumor phenotype within the TME while resisting immunosuppressive signals. Ideally, CAR‐M systems should remain selectively activated at tumor sites and sustain an M1‐like proinflammatory phenotype while maintaining a quiescent or minimally active state in non‐tumor tissues.

To achieve improved precision, safety, and dynamic controllability, substantial efforts have recently been devoted to the development of next‐generation CAR‐M. Rapid advances in synthetic biology have provided powerful engineering tools, including modular genetic circuits, logic‐gated chimeric antigen receptors, inducible expression systems, and synthetic receptors, thereby enabling the rational design and functional optimization of programmable CAR‐M.

#### 3.4.1 Logic‐gated CAR

Logic‐gated CAR systems incorporate Boolean logic operations, such as “OR,” “AND,” and “NOT,” to improve tumor‐targeting specificity while minimizing off‐target toxicity. OR‐gated CAR systems typically consist of two independent activating CARs, in which recognition of either target antigen is sufficient to trigger macrophage activation, thereby improving coverage against tumor antigen heterogeneity.^74^ In contrast, AND‐gated CAR systems require simultaneous recognition of two target antigens to initiate activation and cytotoxic responses, thereby providing an additional safety layer that preserves healthy cells expressing only a single antigen.^75^, ^76^, ^77^ NOT‐gated CAR systems generally combine inhibitory CARs (iCARs) with activating CARs (aCARs), enabling suppression of activation upon recognition of healthy tissue‐associated biomarkers and thereby reducing off‐target toxicity.^78^

#### 3.4.2 Switchable CAR

Switchable CAR is based on modular engineering strategies in which antigen‐recognition domains and signaling domains are physically separated, enabling flexible switching or redirection of antigen specificity without the need to re‐engineer CAR immune cells. This design has led to the development of universal CAR (UniCAR) platforms with enhanced versatility and reprogrammability.^79^ To date, several switchable CAR has been developed, including dimerization platforms based on leucine zipper interactions or biotin–avidin systems, as well as neo‐epitope tagging systems utilizing fluorescein isothiocyanate (FITC), 5B9, or PNE motifs.^80^ Owing to their modularity and adaptability, UniCAR platforms exhibit substantial translational potential for personalized immunotherapy applications.

#### 3.4.3 Microenvironment‐responsive CAR

The immunosuppressive TME in solid tumors is commonly characterized by hypoxia and acidosis, with acidic conditions primarily driven by aberrant metabolism and lactate accumulation.^81^ Based on these features, hypoxia‐responsive or lactate‐activated CAR immune cells have been engineered to remain transcriptionally silent in normal tissues and become selectively activated only within the acidic tumor microenvironment.^82^, ^83^ Similarly, Cao et al. developed inflammation‐responsive CAR‐M systems that can be activated by inflammatory cytokines in damaged tissues. As inflammation resolves, the M2‐like phenotype gradually diminishes, resulting in CAR‐M functions that dynamically depend on the intensity of the inflammatory microenvironment.^15^

#### 3.4.4 Safety switches

Incorporation of safety switches into CAR architectures enables dynamic regulation of CAR‐M activity in vivo, allowing activation under specific pathological conditions or biomarker signals while permitting timely deactivation before severe adverse events occur. Regulatory systems based on proteolysis‐targeting chimeras (PROTACs), small molecules, and light‐dependent transcriptional control have been developed to achieve spatially and temporally restricted CAR activation, thereby minimizing off‐tumor toxicity.^84^, ^85^, ^86^ In addition, suicide switches, including iCasp9, herpes simplex virus thymidine kinase (HSV‐TK), and epitope‐tag‐based elimination systems, can selectively eliminate engineered immune cells in the event of severe toxicity.^87^ Collectively, reversible and controllable switch systems may provide safer, more effective, and clinically translatable strategies for CAR‐based cellular immunotherapy.

## 4 UNIQUE ADVANTAGES OF CAR‐M IN CANCER THERAPY

### 4.1 Comprehensive anti‐tumor actions of CAR‐M

Macrophages are the dominant immune infiltrate in many solid tumors, comprising up to half of all cells.^88^ As highly plastic TAMs, they span M1‐like to M2‐like states with divergent functions. Early lesions often feature M1‐like TAMs that phagocytose cancer cells and release pro‐inflammatory cytokines/chemokines, thereby priming CD4^+^ T helper cells and licensing cytotoxic CD8^+^ T cells. With progression, M2‐like programs prevail, suppressing anti‐sufficient immunity and fostering angiogenesis, especially in invasive disease. Therapeutic reprogramming of TAMs toward an inflammatory, antitumor M1‐like phenotype has therefore emerged as a key objective. Distinct from CAR‐T cells' primarily contact‐dependent cytotoxicity, CAR‐macrophages deploy a multi‐pronged repertoire: phagocytosis, direct cytotoxic activity, cross‐priming and amplification of adaptive responses, and broad remodeling of the tumor microenvironment (Table 1; Figure 4).

**FIGURE 4:**
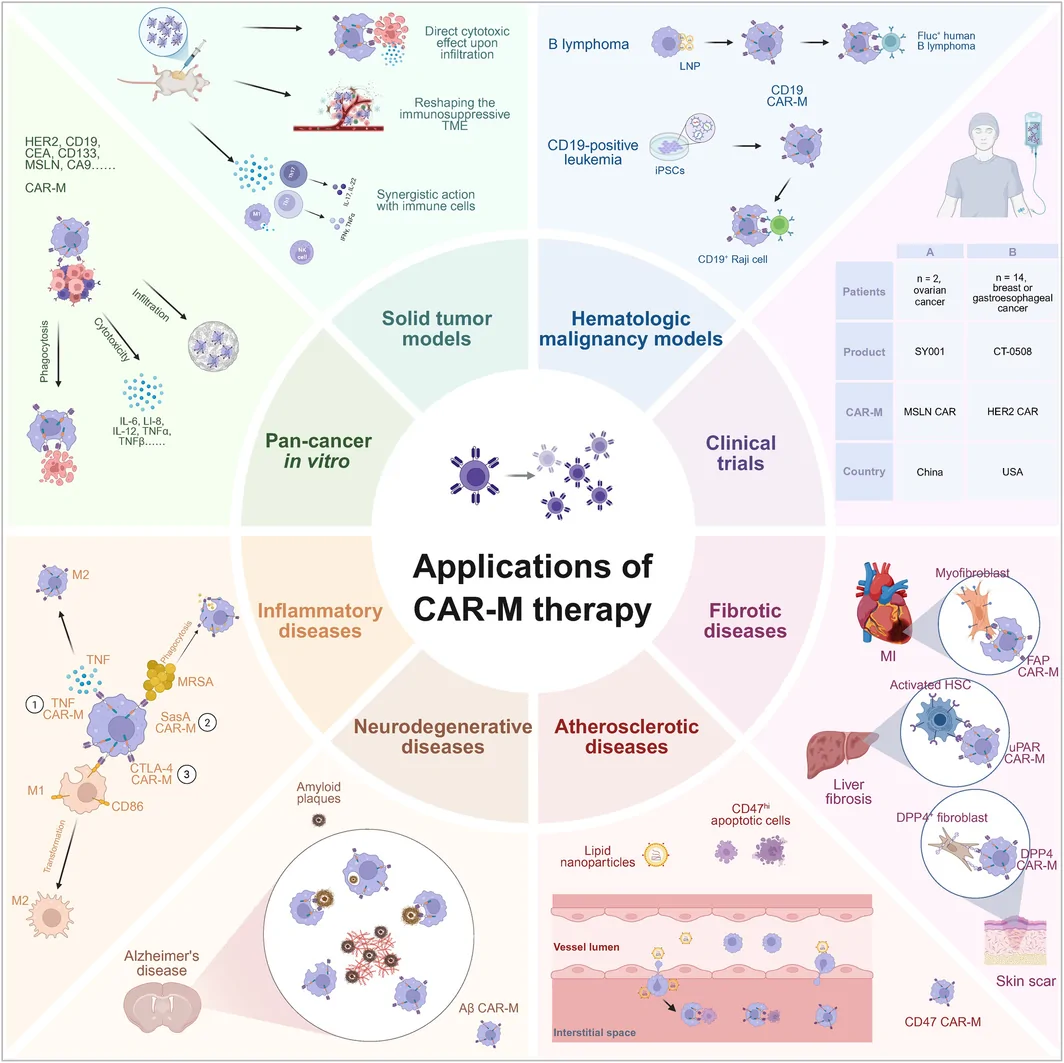
Applications of CAR‐M therapy in cancer and other diseases. Overview of applications of CAR‐M therapy in human diseases, categorized by disease type, including cancer, hematological, fibrotic, atherosclerotic, neurodegenerative, and inflammatory diseases. Created with BioRender.com.

A defining feature of CAR‐M therapy is its capacity to mediate antigen‐specific phagocytosis through chimeric antigen receptors, thereby enabling the engulfment and degradation of tumor cells. A typical CAR construct incorporates intracellular signaling domains such as CD3ζ, FcRγ, IFN‐γ, or FRP5 Mζ, which activate phagocytosis‐related signaling pathways upon recognition of tumor antigens.^33^, ^35^, ^89^, ^90^ At the same time, these domains can enhance proinflammatory M1‐associated pathways, including Toll‐like receptor, tumor necrosis factor (TNF), and IL‐17 signaling, thereby augmenting antitumor immunity.^28^, ^32^ Gao et al. synthesized a universal DNA nanocarrier to introduce an ErbB2‐specific CAR into intratumoral macrophages, reprogramming M2‐type TAMs toward an antitumor M1 phenotype and thereby achieving sustained clearance of invasive tumor cells.^91^ In addition, CAR‐M constructs generated in combination with glycometabolic engineering can strengthen the interactions between macrophages and tumor cells through bioorthogonal reactions, which improves phagocytic efficiency and promotes the release of cytokines such as TNF‐α and IL‐6.^92^

By expressing engineered CAR constructs, CAR‐M can specifically recognize tumor‐associated antigens (TAAs) and activate downstream intracellular signaling pathways, thereby directly mediating macrophage‐mediated cytotoxicity against tumor cells.^51^ During antigen recognition, CAR‐M can secrete proinflammatory cytokines such as IL‐6 and TNF‐α and produce ROS and NO, which induce apoptosis and necrosis of tumor cells.^62^, ^93^ This process further promotes the sustained release of proinflammatory mediators by macrophages, creating a positive feedback loop that continuously amplifies the clearance of tumor cells.

CAR‐M activates adaptive antitumor immune responses through multiple mechanisms. CAR‐M promotes intratumoral infiltration of CD8^+^ cytotoxic T lymphocytes (CTLs) and CD4^+^ helper T cells, and it processes and presents target cell antigens via major histocompatibility complex (MHC) molecules, thereby effectively activating CD8^+^ and CD4^+^ T‐cell responses, initiating systemic antitumor immunity and establishing durable immune memory.^14^, ^94^ At the structural level, CARs that incorporate an FcRγ signaling domain and are engineered to coexpress a secreted CD47 blocker can activate CD8^+^ T cells in situ through a phagocytosis‐dependent cross‐presentation mechanism, further enhancing their proliferation and effector functions.^49^ Interestingly, Jiang et al. used CAR‐M‐derived CAR exosomes as targeted delivery vehicles for high concentrations of CXCL10 and developed a novel drug conjugate termed CAR EDC, which markedly enhances T lymphocyte activation and migratory capacity and promotes their differentiation toward CD8^+^ T cells.^95^ In addition, CAR‐M can recruit multiple immune cell populations, including NK cells and dendritic cells, and it enhances their activity through the secretion of proinflammatory cytokines, thereby synergistically improving antitumor efficacy and overall survival.^20^, ^30^, ^37^

CAR‐M can remodel the tumor microenvironment through multiple mechanisms. Upon activation, CAR‐M upregulates chemokines such as CXCL9 and CXCL10 and cytokines including IL‐1β, TNF‐α, MCP‐1 and IL‐6, which promote intratumoral lymphocyte infiltration and inhibit the migration of immunosuppressive cells.^13^, ^96^ In pancreatic ductal adenocarcinoma models, CAR‐M not only phagocytoses FAP^+^ tumor‐associated fibroblasts but also markedly reduces collagen matrix deposition, thereby alleviating the degree of fibrosis in PDAC.^97^ A similar mechanism has been demonstrated in breast cancer, where CAR‐M activated through recognition of HER2 tumor antigens triggers CD147 inward signaling and upregulates multiple matrix metalloproteinases, including MMP3, MMP14, and MMP15. This reduces extracellular matrix deposition and facilitates T‐cell infiltration into tumor regions.^98^ These effects help to modulate the tumor microenvironment and substantially enhance the penetration and accumulation of chemotherapeutic agents, immune checkpoint inhibitors and other immune effector cells within tumor tissues. In addition, CAR‐M can suppress the secretion of proangiogenic factors such as VEGFA, FGF2, and ANGPT, thereby restricting tumor angiogenesis and further inhibiting tumor growth.^99^

### 4.2 Broad applicability of CAR‐M therapy across solid tumors

CAR‐T cell therapy has achieved remarkable success in the treatment of hematologic malignancies, yet its application in solid tumors still faces substantial obstacles. In contrast, macrophages possess superior tumor‐infiltrating capacity and exert multiple antitumor functions, including phagocytosis, antigen presentation, and the secretion of proinflammatory cytokines, all of which play a central role in the elimination of tumor cells. On this basis, CAR‐M therapy is expected to overcome the delivery barriers encountered by CAR‐T cells in solid tumors and to counteract immune suppression and target antigen heterogeneity within the tumor microenvironment, thereby promoting the extension of CAR‐based strategies into the field of solid tumor therapy.

At present, multiple clinical trials of CAR‐M therapies have been initiated worldwide. Representative examples include the HER2‐targeted CAR‐M candidate CT‐0508 for the treatment of breast cancer and gastric/gastroesophageal junction cancers (NCT04660929); a similar HER2‐directed CAR‐M product for breast cancer (NCT05007379) and advanced gastric cancer with peritoneal metastases (NCT06224738); a mesothelin (MSLN)‐targeted CAR‐M for the treatment of advanced or metastatic solid tumors with high MSLN expression (NCT05164666); the mesothelin‐targeted CAR‐M candidate MCY‐11 for ovarian cancer and peritoneal mesothelioma (NCT03608618), as well as a related mesothelin‐directed therapy for advanced solid tumors (NCT06562647); and a GPC3‐targeted CAR‐M for the treatment of advanced solid tumors (NCT04405778).^8^, ^20^, ^37^

In addition, extensive preclinical studies in cell‐based and animal models have demonstrated that CAR‐M therapy exhibits promising anti‐tumor activity across a wide range of malignancies (Table 1).

### 4.3 Combination strategies of CAR‐M in antitumor therapy

The combination of CAR‐M therapy with chemotherapy, immune checkpoint inhibitors (ICIs), anti‐CD47 agents, and CAR‐T cell therapy has the potential to synergistically enhance antitumor efficacy. As a core modality for advanced and adjuvant treatment of malignancies, cytotoxic chemotherapy can interact with CAR‐M through multiple cooperative mechanisms. Chemotherapeutic agents induce immunogenic cell death and promote the release of TAAs or tumor‐specific antigens (TSAs), which in turn recruit macrophages to phagocytose cellular debris or residual tumor cells and thereby accelerate the reduction in tumor burden.^100^ At the same time, CAR‐M can increase the intratumoral penetration of chemotherapeutic drugs such as gemcitabine (GEM), SN‐38 and oxaliplatin, enhance the sensitivity of tumor cells to chemotherapy and significantly prolong survival in animal models.^95^, ^97^, ^101^ In addition, the combination of CAR‐M with chemotherapeutic agents can further promote macrophage polarization toward an antitumor M1 phenotype, providing a new direction for the development of cancer treatment strategies.

ICIs, particularly anti PD‐1/programmed death ligand 1 (PD‐L1) therapies, have markedly improved treatment outcomes in multiple cancer types, and nearly half of patients can achieve tumor regression and durable tumor control.^102^ Preclinical studies have shown that combining CAR‐M with ICIs can further inhibit tumor growth, enhance remodeling of the tumor microenvironment, and prolong survival.^89^, ^94^ CD47 is an immunoregulatory protein that is highly expressed in many tumors and delivers a “don't eat me” signal by interacting with SIRPα on the surface of macrophages, thereby suppressing phagocytosis.^103^ Overexpression of CD47 enables tumor cells to evade innate immune surveillance. However, blockade of CD47 alone is not sufficient to fully activate the antitumor functions of macrophages, which still require the antigen‐specific “eat me” signals provided by CAR‐M. The combination of CAR‐M therapy with anti CD47 approaches can markedly enhance the phagocytic activity of TAMs and promote the activation of adaptive anti‐tumor T‐cell responses and neoantigen specific T‐cell immunity.^35^ This combinatorial strategy has shown pronounced efficacy in animal models of glioblastoma and hepatocellular carcinoma, which provides experimental support for clinical translation.^39^, ^44^, ^104^

CAR‐M and CAR‐T therapies exhibit synergistic cytotoxic effects in cancer treatment. This synergy may depend on an immune feedback circuit initiated upon CAR‐T cell activation, in which inflammatory cytokines released by CAR‐T cells upregulate the expression of costimulatory ligands such as CD86 and CD80 on CAR‐M, thereby driving macrophage polarization toward an M1 phenotype and enhancing their cytotoxic functions. At the same time, activated CAR‐M further promotes the adaptive immune responses and activation status of CAR‐T cells through antigen presentation and cytokine release, establishing a positive regulatory loop that markedly strengthens their combined tumoricidal activity.^29^, ^105^ In addition, CAR‐M can degrade the dense extracellular matrix within tumor tissues, thereby facilitating CAR‐T cell infiltration and improving overall antitumor efficacy.^30^ Studies have also shown that combining CAR‐M with circular RNA (circRNA) vaccines can synergistically enhance antitumor immune responses in multiple mouse models, further expanding the therapeutic potential of this strategy.^106^

## 5 APPLICATIONS OF CAR‐M THERAPY BEYOND CANCER

### 5.1 CAR‐M therapy in infectious diseases

In infectious diseases, macrophages are front‐line defenders against pathogens, yet organisms such as *S. aureus* can subvert macrophage phagocytic immunity.^107^, ^108^ In a hematogenous *S. aureus* implant‐associated infection model in mice, Ziyang et al. used an implantable nanoparticle coating to generate SasA‐targeted CAR‐M at the peri‐implant site and achieved marked reduction of infection.^109^ In a murine sepsis model, Chunwei et al. developed CRV peptide‐modified lipid nanoparticles encapsulating SasA CAR mRNA (CRV/LNP‐RNA) to enable transient in situ editing of macrophages, which promoted efficient phagocytosis and clearance of methicillin‐resistant *S. aureus*.^57^ In work relevant to coronavirus disease 2019, Wenyan et al. engineered CR3022‐based CAR‐M that recognize the SARS‐CoV‐2 spike protein; in vitro these cells effectively engulfed viral particles and spike‐infected cells.^110^

### 5.2 CAR‐M therapy in inflammatory diseases

Macrophages activated within specific microenvironments are often categorized into two phenotypic classes, classically activated proinflammatory M1 and alternatively activated anti‐inflammatory and pro‐repair M2.^111^, ^112^ They play essential roles in the resolution of inflammation, and depletion of macrophages can cause profound defects in tissue repair.^113^, ^114^ Yu et al. developed a light‐responsive system termed UZPM that was fused with liposomes and introduced into macrophages to generate CTLA‐4 CAR‐M targeting CD86. These cells efficiently traversed the blood–brain barrier, selectively restrained microglial M1 polarization, and prevented the development of inflammation‐associated depression.^43^ In a separate study, Qi et al. engineered CAR‐M that recognize TNF‐α and simultaneously activate intracellular IL‐4 signaling. This configuration achieved anti‐inflammatory activity together with tissue repair and demonstrated therapeutic efficacy in acute and chronic inflammatory models of liver and kidney injury in mice.^15^

### 5.3 CAR‐M therapy in fibrotic diseases

Dysregulated inflammation and tissue repair lead to fibrosis or scarring, a common outcome of chronic diseases characterized by excessive extracellular matrix deposition.^115^, ^116^ Macrophage‐derived transforming growth factor β1 (TGF‐β1) is among the most potent profibrotic mediators.^117^ Activation of fibroblasts is another hallmark of fibrotic pathology.^118^ In a mouse model of myocardial ischemia–reperfusion injury, Moshi et al. engineered fibroblast activation protein (FAP)‐targeted CAR‐M that reduced cardiac fibrosis by phagocytosing activated fibroblasts.^119^ They further developed lipid nanoparticles encapsulating FAP CAR mRNA to reprogram macrophages in situ after myocardial infarction, which attenuated cardiac fibrosis.^16^ Another study co‐delivered mRNAs encoding the lysosomal cysteine protease legumain (Lgmn) and a FAP CAR to generate a fibrosis‐focused CAR‐M with enhanced phagocytic activity.^42^ In addition, CAR‐M directed to fibroblast‐associated markers dipeptidyl peptidase 4 (DPP4) and uPAR effectively mitigated skin scarring and hepatic fibrosis in mouse models.^120^, ^121^

### 5.4 CAR‐M therapy in Alzheimer's disease

Deposition of β‐amyloid (Aβ) plaques is a key driver of Alzheimer's disease, and reducing amyloid burden can meaningfully improve cognition.^122^, ^123^ Alexander et al. engineered Aβ‐targeted CAR‐M. First‐generation Aβ CAR‐M absorbed soluble Aβ and amyloid plaques in vitro and took up and degraded Aβ deposits in brain slices from APP/PS1 transgenic mice, but they showed limited survival in vivo and did not significantly reduce local plaque load. A subsequent “reinforced” CAR‐M configuration that secretes M‐CSF to support its own persistence achieved robust in vivo survival and significantly lowered amyloid plaque levels in mice.^65^

### 5.5 CAR‐M therapy in atherosclerotic disease

Macrophage‐mediated efferocytosis is essential for clearing apoptotic cells within atherosclerotic lesions and thereby limiting inflammation. Up‐regulation of CD47, a canonical “don't eat me” signal, confers resistance to macrophage phagocytosis.^124^, ^125^ To overcome this barrier, Skylar et al. engineered CD47‐targeted CAR‐M to engulf CD47^Hi^ apoptotic cells, then refined the platform with a reactive oxygen species (ROS)–responsive lipid nanoparticle that modified CD47 CAR‐M to activate liver X receptor signaling and enhance lipid catabolism. This configuration increased cholesterol efflux and curtailed overall lipid burden while augmenting CAR‐M phagocytic activity.^126^

## 6 EARLY CLINICAL RESULTS OF CAR‐M THERAPY

As of December 23, 2025, 12 clinical studies of CAR‐M have been identified, including 9 registered on ClinicalTrials.gov and 3 in the Chinese Clinical Trial Registry (Table 4). Most trials enroll patients with solid tumors that overexpress HER2 or mesothelin, with one study targeting CD5^+^ T‐cell lymphoma and another evaluating tumors with high GPC3 expression. CAR‐M products are principally derived from autologous PBMCs and from iPSCs. Routes of administration include intravenous infusion and, in selected cases, intraperitoneal perfusion, depending on the clinical scenario. These studies are predominantly observational or phase I, with primary endpoints focused on safety and tolerability. Two additional studies, ChiCTR2500103658 and NCT06823713, investigate engineered macrophage therapy for liver cirrhosis; because these protocols do not clearly employ CAR‐modified macrophages, they were not included in this review.

**TABLE 4:** Clinical trials of CAR‐M.

No.	ID	Phase	Title	Product name	Target antigen	Disease	Status	Country
1	NCT04660929	I	CAR‐macrophages for the treatment of HER2 overexpressing solid tumors	CT‐0508	HER2	HER2 overexpressing solid tumors	Active, not recruiting	United States
2	NCT05007379	Observational	Cohort study to determine the antitumor activity of new CAR‐macrophages in breast cancer patients' derived organoids	NA	HER2	Breast cancer	Unknown	NA
3	NCT06224738	I	Human HER2‐targeted macrophages therapy for HER2‐positive advanced gastric cancer with peritoneal metastases	NA	HER2	Advanced gastric cancer with peritoneal metastases	Not yet recruiting	China
4	NCT06562647	NA	SY001 targets mesothelin in a single‐arm, dose‐increasing setting in subjects with advanced solid tumors	SY001	MSLN	Advanced ovarian cancer/pancreatic cancer	Recruiting	China
5	NCT06254807	I	CAR‐monocytes for the treatment of HER2 overexpressing solid tumors	CT‐0525	HER2	HER2 overexpressing solid tumors	Active, not recruiting	United States
6	NCT03608618	I	Intraperitoneal MCY‐M11 (mesothelin‐targeting CAR) for treatment of advanced ovarian cancer and peritoneal mesothelioma	MCY‐M11	MSLN	Metastatic or unresectable high‐grade serous adenocarcinoma	Terminated	United States
7	NCT05138458	I/II	A study of MT‐101 in subjects with CD5+ relapsed/refractory TCL	MT‐101	CD5	T‐cell lymphoma	Suspended	United States
8	NCT05164666	I	A study of TAK‐103 in adult with solid tumors	TAK‐103	MSLN	Mesothelin‐expressing advanced or metastatic solid tumors	Terminated	Japan
9	NCT04405778	I	A study of TAK‐102 in adult with previously‐treated solid tumors	TAK‐102	GPC3	Solid tumors with GPC3	Terminated	Japan
10	ChiCTR2500097356	Observational	Construction of chimeric antigen receptor macrophages (CAR‐M) and its application in immunotherapy for malignant tumor xenografts	NA	NA	Gliomas and brain metastases (breast/lung cancer)	Recruiting	China
11	ChiCTR2400082776	NA	Clinical study of CAR‐M cells therapy in solid tumors	NA	HER2	HER2 overexpressing solid tumors	Not yet recruiting	China
12	ChiCTR2400080078	I	Clinical study of CAR‐M cells for the treatment of relapsed/refractory ovarian cancer with high HER 2 expression	NA	HER2	Relapsed/refractory ovarian cancer with high HER 2 expression	Recruiting	China

NCT03608618, the first CAR‐M trial to report results, was presented in an abstract.^52^ Autologous PBMCs were electroporated with MSLN CAR mRNA, then administered intraperitoneally once weekly for three doses using dose escalation. To date, 11 patients with ovarian cancer or malignant peritoneal mesothelioma have been treated across the first three dose levels with favorable safety and tolerability. There were no infusion‐related reactions, no dose‐limiting toxicities, and no neurotoxicity.

NCT05138458, the progress was briefly reported in 2023 as a poster presentation.^17^ This study developed CD5‐directed CAR‐M (MT‐101) for patients with relapsed or refractory T‐cell lymphoma. Among three treated participants, MT‐101 was well tolerated. No cytokine release syndrome, no immune effector cell‐associated neurotoxicity syndrome, and no infusion reactions were observed. One participant achieved an overall survival exceeding 10 months, which is longer than the reported median overall survival of 5.5 months for relapsed or refractory peripheral T‐cell lymphoma.

NCT06562647 is the first CAR‐M clinical trial with peer‐reviewed results.^18^ The study evaluated mesothelin‐directed CAR‐M (SY001) in two patients with advanced ovarian cancer. Transient post‐infusion cytokine fluctuations and lymphopenia or neutropenia were observed in both patients, but no grade ≥3 adverse events and no cytokine release syndrome occurred. At day 28, both patients maintained stable disease, supporting a favorable safety profile for SY001. Notably, the SY001 product infused in participant 2 contained 8.87% T cells, indicating the feasibility of administering a mixed cellular product.

NCT04660929 is the first phase I CAR‐M trial to report human data.^19^, ^20^ The study evaluated a HER2‐directed CAR‐M product (CT‐0508) in fourteen patients with HER2‐overexpressing solid tumors. CT‐0508 was administered by intravenous infusion, with patients assigned to single‐dose or multi‐dose cohorts. Participants were followed for up to 1 year after infusion. No grade 3 or 4 cytokine release syndrome and no immune effector cell‐associated neurotoxicity were observed. Among 13 radiographically evaluable patients, 40% experienced reductions in target lesions after CT‐0508. In 11 on‐treatment biopsy specimens, CT‐0508 was detectable in 92% of cases, and remained detectable in 27% at week 4. Single‐cell RNA sequencing and T‐cell receptor repertoire analyses indicated remodeling of the tumor microenvironment with enhancement of adaptive immunity. Notably, in contrast to many CAR‐T protocols, CT‐0508 did not require lymphodepleting chemotherapy before infusion.

## 7 A CONCEPTUAL “4S” FRAMEWORK FOR NEXT‐GENERATION CAR‐M THERAPY

Since its inception in 2006, CAR‐M technology has produced encouraging anti‐tumor activity in vitro and in animal models and has expanded into non‐oncologic indications, including infectious, fibrotic, and inflammatory diseases, as well as Alzheimer's disease and atherosclerosis (Tables 1, 2; Figure 4). Across the observational and phase I studies that have reported results to date, CAR‐M has shown a favorable safety and tolerability profile, although clinical efficacy remains to be defined more clearly (Table 4). At present, most CAR‐M constructs are first‐ or second‐generation designs that primarily program phagocytosis and, in some cases, cytokine release. Key areas of ongoing work include optimization of cell sources, manufacturing and scale‐up, stability within disease microenvironments, and toxicity management. To organize these priorities, we propose a “4S framework” (specificity, switchability, synergy, and safety), which together capture the central challenges of current CAR‐M research and point to actionable directions for next‐generation development (Figure 5).

**FIGURE 5:**
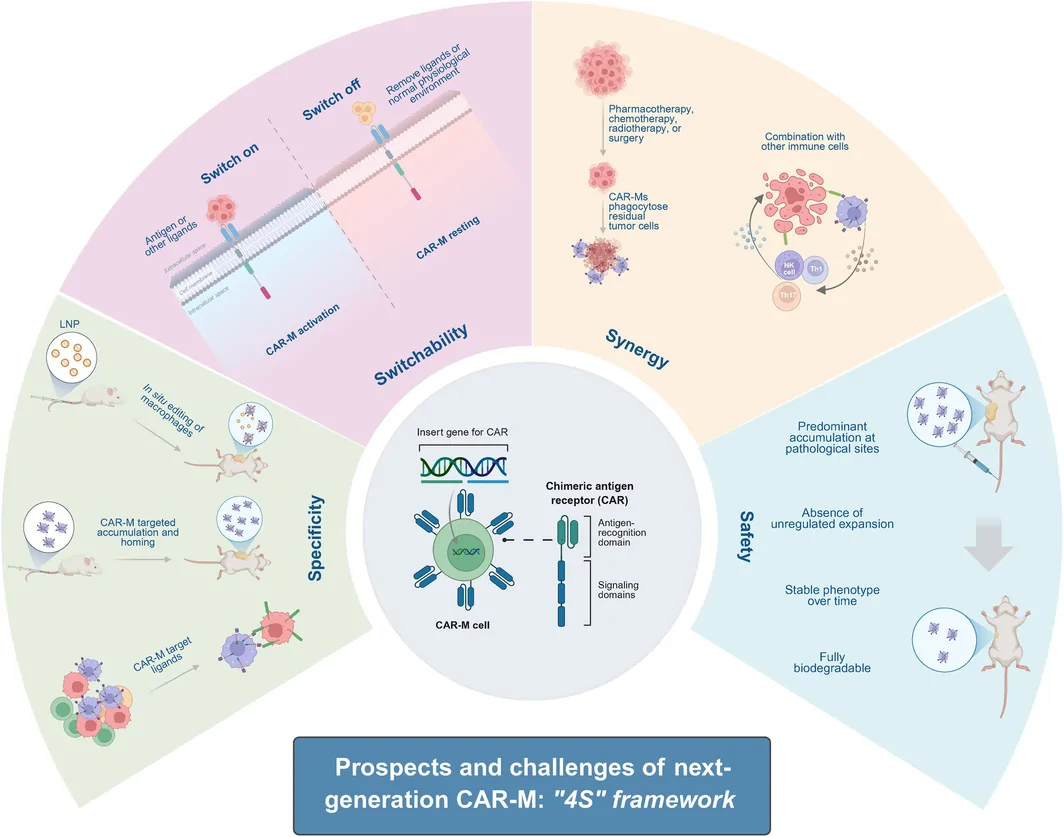
Representative images summarizing prospects and challenges of next‐generation CAR‐M cell therapy. “4S” framework: (1) specificity: precise recognition and active homing of CAR‐M, and in situ editing of macrophages; (2) switchability: activation and resting in CAR‐M cell; (3) synergy: combination therapy of CAR‐M cell therapy and other anticancer agents; (4) safety: persistence, engraftment and stability. Created with BioRender.com.

### 7.1 Specificity

Specificity encompasses two dimensions: active homing of CAR‐M to diseased sites and precise ligand recognition by the CAR scaffold. Macrophages are key sentinels of the innate immune system with strong lesion tropism, high biocompatibility, and in vivo persistence that can extend for months.^127^, ^128^ Their inherent propensity to infiltrate tumors, inflamed tissues, and sites of injury provides a natural advantage for targeted delivery.^129^, ^130^, ^131^ Viral transduction with lentivirus or adenovirus can activate inflammasomes and bias CAR‐M toward a proinflammatory M1 phenotype, which may further improve in vivo trafficking and lesion targeting.^35^, ^49^ In addition, preemptive knockdown of furin with siRNA has been shown to strengthen the proinflammatory phenotype of CAR‐M.^63^ These strategies enhance the localization of CAR‐M to pathological niches. Target recognition is governed primarily by the extracellular single‐chain variable fragment, which confers high‐affinity and high‐specificity binding to tumor antigens.^25^, ^26^ A newer design fuses the extracellular domains of IL‐10 or TGFβ receptors to the intracellular domains of the IFN‐γ receptor.^13^ This configuration exemplifies a CSSR and provides a broadly applicable framework for receptor‐level engineering beyond conventional CAR formats.

In addition, in situ editing of macrophages can be achieved by delivering CAR constructs directly to lesion‐resident cells using nanoparticles or biomembrane‐based carriers. Because this route may increase the risk of off‐target delivery, investigators have optimized both material modifications and administration strategies. Yuchen et al. developed an inhalable engineered small extracellular vesicle platform in which anti‐CD206 scFv was integrated on the vesicle surface, thereby improving the precision of targeting CD206^+^ macrophages in the lung.^50^ Lei et al. used enucleated mesenchymal stem cells as carriers for CAR plasmids. These enucleated cells exhibited active homing to glioblastoma multiforme and underwent apoptosis that preferentially triggered macrophage phagocytosis, which enhanced lesion‐specific uptake.^44^ Additional targeting ligands and materials, including 1,2‐dioleoyl‐sn‐glycero‐3‐phospho‐L‐serine, legumain, the CRV peptide, mannose, hydrogels, and oxidatively modified lipid nanoparticles, have further improved the specificity of macrophage‐directed delivery.^16^, ^42^, ^57^, ^69^, ^97^, ^132^

### 7.2 Switchability

Next‐generation CAR design should enable context‐dependent control: switching on reparative signaling within pathological microenvironments and switching off to maintain quiescence once pathological cues abate or under physiological conditions. At present, the extracellular domains of most CAR‐M constructs are single‐chain variable fragments that target specific antigens. An scFv is an engineered antibody fragment formed by linking the VH and VL coding sequences from the same antibody with a flexible peptide linker to create a single‐chain variable fragment.^133^, ^134^, ^135^ This configuration improves binding specificity, yet key questions remain about how intracellular signaling is effectively initiated in macrophages and whether such signal transduction depends on antigen concentration and the duration of engagement. Analogous to CAR‐T, some studies employ CD3ζ‐based signaling modules in CAR‐M to activate phagocytosis and anti‐tumor functions.^94^, ^97^, ^136^ In monocytes and macrophages, however, endogenous CD3ζ expression at the membrane is limited. One study showed that signaling components downstream of FcγRI, mediated by integrin α1β1, more effectively controlled enhanced CAR‐M activation and phagocytosis than CD3ζ‐based designs.^32^ These differences may depend on the cellular source used for CAR‐M manufacturing.

Building on CAR‐T research, logic‐gate design can improve the stability of CAR on–off control.^22^ In an AND gate configuration, activation requires recognition of two antigens. Erhao et al. constructed a dual‐input system that combined a CEA–CD3ζ module with an MSLN‐4‐1BB module; cytotoxicity was robust against tumor cells coexpressing both antigens and absent against cells expressing only one antigen.^137^ In a separate SynNotch CAR‐T approach, effector activity occurred only after prior triggering by a synNotch ligand, which then induced CAR‐directed killing.^138^, ^139^ A NOT gate can attenuate CAR‐T cytotoxicity in the presence of a safety antigen and thereby improve selectivity. TROP2 is highly expressed in epithelial cancers but is also present in normal tissues of kidney, lung, and skin.^140^, ^141^ Nathanael et al. coupled a TROP2 scFv to the inhibitory PD‐1 signaling domain and coexpressed this construct with a CEACAM5 CAR in T cells; engagement of TROP2 on normal cells suppressed killing by the CEACAM5 CAR.^142^ These multi‐antigen schemes illustrate complex signal crosstalk that could be adapted to CAR‐M, although macrophage‐specific signaling thresholds, timing, and phenotypic outcomes require further evaluation.^143^

Another strategy is to engineer CSSR. Unlike scFv‐based CARs, the extracellular sensing domain of a CSSR is built from endogenous receptor architecture. Sabrina et al. created CSSR‐M by fusing the extracellular domains of the IL‐10 or TGF‐β receptors to the intracellular domains of the IFN‐γ receptor, thereby converting immunosuppressive cues into pro‐inflammatory, anti‐tumor signaling.^13^ In physiological tissues, basal amounts of IL‐10 or TGF‐β1 are present but remain below the threshold needed to activate macrophages through this design. This implies that, once pathological signals abate or in normal microenvironments, CSSR‐M reverts to a quiescent state. By contrast, scFv‐based CARs may be prone to sustained or misdirected activation and, because they contain exogenous antibody fragments, may also elicit anti‐drug immune responses.

### 7.3 Synergy

As noted above, in preclinical studies the combination of CAR‐M therapy with chemotherapy, ICIs, anti‐CD47 approaches and CAR‐T cell therapy has shown the potential to synergistically enhance anti‐tumor efficacy. Within the complex TME, reliance on a single modality may not be the optimal strategy. Preserving the cytotoxic activity of T cells and NK cells is beneficial for inducing immunogenic cell death and the release of TAAs, which in turn augments CAR‐M‐mediated phagocytosis, antigen presentation and proinflammatory cytokine secretion.^22^, ^100^, ^144^ At the same time, inhibition or blockade of immune checkpoints and CD47 can prevent T‐cell exhaustion and overcome CAR‐M phagocytic impairment.^35^, ^49^, ^145^ In addition, combination with conventional approaches such as surgical resection and radiotherapy also represents a potential therapeutic strategy.

### 7.4 Safety

Early CAR‐M trials have demonstrated good tolerability, yet several safety dimensions warrant attention in future applications. First is off‐target toxicity. Systemic administration distributes CAR‐M widely, particularly to liver, lung, and kidney, which may precipitate CRS.^15^, ^20^, ^73^ Even lesion‐directed CAR‐M can potentially damage normal tissues, and in situ editing strategies that deliver CAR via biomaterials may further increase off‐target risk. Second is phenotypic stability. An ideal CAR‐M activates within pathological contexts and remains quiescent once removed from disease cues or under physiological conditions; such switchability is central to CAR design. Third is controllability and degradation. A biodistribution study showed that CAR‐M retained functional activity for up to 21 days in tumor‐bearing mice and persisted in peripheral blood, lung, and liver for as long as 60 days.^89^ Incorporation of an inducible caspase‐9 suicide switch enables pharmacologic control of CAR‐M lifespan with AP1903.^89^ Additional control modalities developed in CAR‐T, including protease‐regulated platforms and focused ultrasound, may also be adapted to regulate CAR‐M activity.^146^, ^147^, ^148^ Finally, although macrophages are terminally differentiated, iPSC‐ or progenitor‐derived CAR‐M may carry risks of tumorigenicity or aberrant proliferation and therefore require rigorous qualification.

## 8 CONCLUSION

Compared with the challenges faced by conventional CAR‐T therapy in solid tumors, including poor infiltration into tightly structured tumor masses, immunosuppression within the TME, and marked antigen heterogeneity, CAR‐M shows substantial therapeutic promise and represents a leading edge in cancer immunotherapy. Across preclinical studies, CAR‐M demonstrates targeted phagocytosis of tumor cells, secretion of proinflammatory mediators that tune innate immunity, and secondary activation of cytotoxic T lymphocytes. Although current clinical experience remains limited and largely confined to observational and phase I trials, the reported data support a favorable safety and tolerability profile. Important gaps remain in manufacturing workflows, patient treatment algorithms, measures of efficacy, and standardized endpoints for response assessment, which will require rigorous clinical validation. Beyond oncology, encouraging results in infectious, inflammatory, and fibrotic diseases, as well as atherosclerosis and Alzheimer's disease, suggest broad applicability and a large development horizon for CAR‐M therapies.

In summary, this review systematically summarizes the design principles of CAR‐M, with a particular emphasis on emerging engineering strategies for next‐generation CAR‐M as well as evidence from preclinical studies and clinical trials. Although CAR‐M therapy remains in its early stages, it offers substantial opportunities for exploration. To facilitate translation from bench to bedside, we propose a next‐generation “4S framework” (specificity, switchability, synergy, and safety) for CAR‐M development that addresses key barriers to clinical application.

This manuscript is a narrative review article and does not involve any original experimental work. No cell lines, primary cells, or animal or human tissues were generated or used by the authors in this study. Therefore, information regarding species, sex, tissue of origin, cell line authentication, RRIDs, mycoplasma testing, or contamination status is not applicable and does not affect the conclusions of this review.

## AUTHOR CONTRIBUTIONS

**Heng Wang:** Conceptualization; methodology; data curation; formal analysis; software; writing – original draft. **Guoping Zheng:** Conceptualization; supervision; funding acquisition; writing – review and editing. **Yun Zhou:** Methodology; writing – review and editing. **Yaling Li:** Conceptualization; methodology; data curation; formal analysis; software; writing – original draft. **Yiwei Shi:** Methodology; writing – review and editing.

## CONFLICT OF INTEREST STATEMENT

The authors declare no competing interests.

## Data Availability

Data sharing not applicable to this article as no datasets were generated or analyzed during the current study.
